# Physically Defective Children

**Published:** 1923-03

**Authors:** 


### PHYSICALLY DEFECTIVE CHILDREN.

The London County Council has just published (P. S. King & Co., 9d.) the report on Physically Defective Children, made by Major R. C. Elmslie, M.B., &c., to Sir W. H. Hamer, its School Medical Officer. Major Elmslie visited the whole of the schools for children, 2,891 in number, to whom this description applies, and tabulates the diseases and deformities from which they were suffering. The first noticeable fact in a comparison of these figures with those of 1907 and 1914 is the diminution in the number of children suffering from deformities due to tuberculous disease, and the increase in the number suffering from infantile paralysis. This does not necessarily indicate a diminished incidence of the first of these diseases or an increased incidence of the second. In examination of the individual cases it is noticeable that the physical condition of the children suffering from tuberculous disease is decidedly better than it was at the time of the previous examinations.

### Defective Treatment of Infantile Paralysis.

Major Elmslie's remarks on the treatment of infantile paralysis are exceedingly disquieting. He writes :—

The treatment of infantile paralysis in London at the present time is very defective, and a better plan for attacking this, the most serious cause of crippling in children, is urgently needed. In the first place, I suggest that it would be very valuable to secure definite accommodation in some one institution for the treatment of infantile paralysis in its early stages. Provision for infants of under one year of age is essential, and it should be arranged that patients could remain in the hospital for periods of up to two or three years, if necessary. A hospital specialising in the treatment of infantile paralysis should preferably be just outside London, and must be fully equipped for splinting, massage, electrical work, make its own surgical appliances, and have facilities for operating; in fact, it must be a fully equipped orthopædic hospital. In order to secure continuity of treatment, which is one of the essentials of these cases, it should be linked up with the out-patient departments of those London hospitals at which the major part of the orthopædic work is carried out.

### Valueless Recommendations.

Major Elmslie doubts whether the schools for physically defective children "are doing as much as they might" as centres for the care of cripples, and uses severe language about their medical organisation :—

In my opinion, the special medical work in these schools should be carried out by surgeons and physicians who are actively occupied in the treatment of similar cases. During the war period the routine examinations have had to be carried out chiefly by whole-time medical officers on the general service of the Council. Naturally, the knowledge that these medical officers possess of the treatment of crippling deformities is small, and they take no part in the treatment of these conditions. Their recommendations are, for this reason, seldom of any value, and if they make any definite recommendations, they are liable to offend hospital surgeons by suggesting lines of treatment which are inadvisable or incorrect. I think that care should be taken that re-examinations are not designed simply to satisfy formally the requirements of the Board of Education, but that they should aim at a careful investigation of the children's condition with a view to suggesting any amelioration of it, if possible. It also appears to me that the care of the children in the physically defective schools, so far as provision of treatment, care of instruments, and co-ordinated with hospitals is concerned, is not, on the whole, as good as it was in 1914.

